# Which sagittal evaluation system can effectively predict mechanical complications in the treatment of elderly patients with adult degenerative scoliosis? Roussouly classification or Global Alignment and Proportion (GAP) Score

**DOI:** 10.1186/s13018-021-02786-8

**Published:** 2021-10-26

**Authors:** Xiangyao Sun, Wenzhi Sun, Siyuan Sun, Hailiang Hu, Sitao Zhang, Chao Kong, Shibao Lu

**Affiliations:** 1 Department of Orthopaedics, Xuanwu Hospital Capital Medical University, No. 45 Changchun Street, Xicheng District, Beijing, 100053 China; 2 National Clinical Research Center for Geriatric Diseases, Beijing, 100053 China; 3 Department of Interdisciplinary, Life Science, Purdue University, West Lafayette, IN 47907 USA

**Keywords:** Adult degenerative scoliosis, Roussouly classification, Global Alignment and Proportion Score, Mechanical complications, effectiveness

## Abstract

**Background:**

To achieve the proper sagittal alignment, previous studies have developed different assessment systems for adult degenerative scoliosis (ADS) which could help the spine surgeon in making treatment strategies. The purpose of our study is to evaluate whether Roussouly classification or global alignment and proportion (GAP) score is more appropriate in the prediction of mechanical complications after surgical treatment of ADS.

**Methods:**

ADS patients who received long segmental fusion in the treatment during the period from December 2016 to December 2018 were evaluated in this study. Basic information and radiologic measurements were collected for analysis. Patients were divided into two groups according to occurrence or absence of mechanical complications for comparison. Mechanical complications included proximal junctional kyphosis (PJK), proximal junctional failure (PJF). GAP categories divided GAP score into proportioned spinopelvic position, moderately disproportioned position, and severely disproportioned position according to the cut-off values. The correlation between evaluation systems and mechanical complications was analyzed through a logistic regression model via stepwise backward elimination based on the Wald statistics. Receiver operator characteristic (ROC) curve was used to determine the predictability of the evaluation systems in the occurrence of mechanical complications and calculate their cut-off value. Area under the curve (AUC) was used to evaluate the validity of the thresholds.

**Results:**

A total of 80 patients were included in this study. There were 41 patients in mechanical complication group and 39 patients in no mechanical complication group. GAP score (*P* = 0.008) and GAP categories (*P* = 0.007) were positively correlated with mechanical complications; Roussouly score was negatively correlated with mechanical complications (*P* = 0.034); GAP score was positively correlated with PJK (*P* = 0.021); Roussouly score was negatively correlated with implant-related complications (*P* = 0.018); GAP categories were correlated with implant loosening (*P* = 0.023). Results of ROC showed that GAP score was more effective in predicting PJK (AUC = 0.863) and PJF (AUC = 0.724) than Roussouly score; GAP categories (AUC = 0.561) was more effective than GAP score (AUC = 0.555) in predicting implant-related complications.

**Conclusions:**

Roussouly classification could only be a rough estimate of optimal spinopelvic alignment. Quantitative parameters in GAP score made it more effective in predicting mechanical complications, PJK and PJF than Roussouly classification.

**Supplementary Information:**

The online version contains supplementary material available at 10.1186/s13018-021-02786-8.

## Introduction

Three-dimensional deformity occurs in patients with adult degenerative scoliosis (ADS). Coronal correction of frontal deformity was the principle concerned in the past; ADS was found to be deeply affected by the rotational thoracolumbar kyphosis which could alter the sagittal profile [1]. Previous studies showed that the postoperative complication rates (8.4–42%), revision rates (9–17.6%) in ADS were still high, and could increase after long-term follow-up [2, 3]. Increased junctional stress concentration might cause the collapse of the implant, or vertebra, which could cause mechanical complications such as PJK, distal junction kyphosis (DJK), pseudoarthrosis, rod breakage or vertebral fracture [4–7]. Nowadays, more attention is paid to sagittal deformity. It was reported that spinal degeneration could decrease lumbar lordosis, increase thoracic kyphosis, change the ideal sagittal alignment [8]. To achieve the proper sagittal alignment, previous studies have developed different evaluation systems for degenerative spinal deformity which could help surgeons in making treatment strategies, such as Scoliosis Research Society (SRS)-Schwab classification [9], Roussouly classification [10] and Global Alignment and Proportion (GAP) Score [4].

According to SRS-Schwab classification [9, 11], three targets for corrective surgery realignment are suggested: the pelvic incidence (PI) minus lumbar lordosis (LL) mismatch of less than 10°; pelvic tilt (PT) of less than 20°; sagittal vertical axis (SVA) of less than 4 cm. However, even after matching the targets of Schwab criteria, the mechanical complication rates remain very high (31.7%); this classification is not effective neither in making the treatment strategy nor in predicting clinical outcome, especially when there is no sagittal malalignment [12].

In Roussouly classification, 4 types of spinal alignments were described depending on sacral slope (SS) and the shape of LL. This classification was subsequently updated to a modified classification which included a new type, the anteverted type 3 [13]. This new type was characterized by low-grade PI, SS > 35°, and low or negative PT [13]. All radiographic factors were compared with ideal spinal alignment to evaluate their deviations from the ideal parameters. In addition, the optimal sagittal alignment was determined on the rate of PI in proportion to these factors. This is because PI is an unchanged parameter [4]. Roussouly classification contributes to the determination of high local stress zones in the whole spine. In this classification, the lower the lumbar lordosis or flat back, the higher the stress is on the disks; the more the lumbar lordosis increased, the more is the contact force on the posterior column [5]. Roussouly classification may help the surgeon to predict the best rod bending and the best correction degrees to achieve optimal results. However, degenerative spine modifies the organization of spinal curves which is responsible for the compensation mechanisms at the spine level or in the pelvis, hips, and knees. This can make it difficult to use Roussouly classification in degenerative conditions [14].

Apart from Roussouly classification to help to make surgical strategies, GAP score is an alternative that uses PI-based sagittal parameters to quantify the shape and alignment of the sagittal plane. Both Roussouly classification and GAP score share similar principles to achieve the optimal spinopelvic alignment which includes the restoration of ideal LL, ideal pelvic version, and ideal lordosis distribution [4]. Planning surgical targets in the sagittal plane based on the proportional indices via the GAP score can decrease the occurrence of mechanical complications [7]. However, no study has compared the effectiveness of these two evaluation systems in predicting mechanical complications after long segmental fusion in the treatment of ADS. Therefore, the purpose of our study is to evaluate whether Roussouly classification or GAP score is more appropriate in the prediction of mechanical complications in the treatment of ADS.

## Methods

### Patients selection

Charts of ADS patients who received long segmental fusion during the period from December 2016 to December 2018 were retrospectively included in this study. Basic information of the patients, such as gender, age, body mass index (BMI), follow-up time, blood loss, operation time, vertebrae fused, visual analogue scale (VAS), Japanese Orthopaedic Association (JOA), Oswestry Disability Index (ODI) were collected. Inclusion criteria included: age > 60 years at the time of attendance; more than 4 vertebral levels fused; coronal Cobb angle (CA) ≥ 20°, SVA ≥ 5 cm, PT ≥ 25°, thoracic kyphosis (TK) ≥ 60°, and a follow-up time of more than 2 years. Exclusion criteria included: previous spinal fusion; ADS secondary to syndromic, autoimmune, infectious, tumor, or other pathologic conditions. Written informed consents were signed by all the included patients. The institutional review board approved this study protocol following the declaration of Helsinki principles.

### Radiographic measurements and scoring

Radiologic measurements, such as PI, PT, SS, thoracolumbar kyphosis (TLK), TK, LL, L4-S1 lordosis, global tilt (GT), SVA, number of vertebrae included in the lordosis (NVL), lumbar sagittal apex (LA) and inflexion point (IP), were recorded at 6 weeks postoperatively (Additional file 1). All radiographs were analyzed by validated software (Surgimap, Nemaris Inc., New York, NY). All data were measured separately by independent researchers (XS and WS). When discrepancies arose, a consensus would be taken after being discussed by the coauthors.

Standard values of parameters for Roussouly types are shown in Additional file 2 [1]. Roussouly modifiers of ADS patients were defined as follows: modifier “0”, patients with ideal profiles; modifier “+”, patients with under-corrected profiles; modifier “++”, patients with over-corrected profiles. The Roussouly modifiers were then statistically weighted as Roussouly score (1 for modifier “0”, 2 for modifier “+” and 3 for modifier “++”).

GAP score ranges from 0 to 13 points. It includes relative pelvic version (RPV), relative lumbar lordosis (RLL), lordosis distribution index (LDI), relative spinopelvic alignment (RSA), and age [4]. The cut-off values of GAP score were as follows: a GAP score of 0 to 2 indicated a proportioned spinopelvic position; a GAP score of 3 to 6 was defined as moderately disproportioned; a GAP score of more than 6 was defined as severely disproportioned (Additional file 3) [4].

### Mechanical complications

The mechanical complications discussed in this study included: proximal junctional kyphosis/ failure (PJK or PJF), distal junctional kyphosis/ failure (DJK or DJF), and implant-related complications [4]. PJK was defined as a kyphosis between upper instrumented vertebra (UIV) and UIV + 2 increased of ≥ 10° in between early postoperative and follow-up radiographs. PJF was the fracture of UIV or UIV + 1, pullout of instrumentation at UIV, and/or sagittal subluxation. DJK or DJF was a postoperative kyphosis angle between lower instrumented vertebra (LIV) and LIV-1 increased of ≥ 10°, and/or pullout of instrumentation at LIV. Implant-related complications were implant loosening, implant breakage, or implant pullout. Patients were divided into mechanical complication group (MC) and no mechanical complication group (NMC).

### Statistical analysis

Statistical analysis was performed using SPSS 17.0 (SPSS Inc, Richmond, CA, USA). Continuous variables were reported as mean ± standard deviations. Kolmogorov–Smirnov test was performed to the normal distribution of the data. Normally distributed values were analyzed with the independent Student t test. Skewed values were analyzed with Kruskal-Wallis test. Categorical variables were reported as the number of cases and compared using Pearson’s Chi-square test. The correlation between evaluation systems and mechanical complications could be found by odds ratio (OR) and 95% confidence interval (CI) in a logistic regression model via stepwise backward elimination based on the Wald statistics. Receiver operating characteristic (ROC) curve was used to determine the predictability of the evaluation systems in the occurrence of mechanical complications and calculate their cut-off value. Area under the curve (AUC) was used to evaluate the validity of the thresholds. A two-tailed *P* value < 0.05 was statistically significant.

## Results

### Demographics

A total of 80 patients were included in this study (Table 1). Mean age was 76.5 ± 2.5 years old. Mean follow-up was 19.3 ± 6.2 months. Implant-related complication (42.5%) had the highest incidence in mechanical complications (51.3%). The most common implant-related complication was implant loosening (37.5%). Postoperative radiographic parameters and clinical scoring systems were significantly improved compared with preoperative data (Table 2).

**Table 1:** Characteristics of the included patients

Variables	Data
Cases	80
Female, n (%)	65 (81.3%)
Age (years)	76.5 ± 2.5
BMI	26.8 ± 3.8
Follow-up time (months)	19.3 ± 6.2
Blood loss (ml)	1052.2 ± 330.0
Operation time (min)	450.9 ± 141.4
Vertebrae fused (n)	6.0 ± 1.9
Mechanical complications, n (%)	41 (51.3%)
PJK, n (%)	5 (6.25%)
PJF, n (%)	2 (2.5%)
DJK or DJF, n (%)	2 (2.5%)
Implant-related complications, n (%)	34 (42.5%)
Implant loosening, n (%)	30 (37.5%)
Implant breakage, n (%)	4 (5%)

Pre, preoperative; Post, postoperative; BMI, body mass index; PJK, proximal junctional kyphosis; PJF, proximal junctional failure; DJK, distal junctional kyphosis; DJF, distal junctional failure

**Table 2:** Radiographic parameters and clinical scores

Items	Pre-data	Post-data	*P* value
CA (°)	22.1 ± 6.9	7.4 ± 2.3	< 0.001
TK (°)	46.2 ± 30.2	30.8 ± 20.1	< 0.001
LL (°)	25.0 ± 14.6	33.9 ± 10.7	< 0.001
SS (°)	24.8 ± 9.5	28.2 ± 7.2	0.024
PT (°)	26.1 ± 14.5	22.3 ± 9.9	0.010
SVA (cm)	9.6 ± 3.7	3.6 ± 3.4	< 0.001
VAS	6.5 ± 1.7	2.7 ± 0.8	< 0.001
JOA score	3.8 ± 1.1	6.1 ± 1.8	< 0.001
ODI	60.0 ± 24.3	26.9 ± 12.8	< 0.001

Pre, preoperative; Post, postoperative; CA, coronal Cobb angle; TK, thoracal kyphosis; LL, lumbar lordosis; SS, sacral slope; PT, pelvic tilt; SVA, sagittal vertical axis; VAS, visual analogue scale; JOA, Japanese Orthopaedic Association; ODI, Oswestry Disability Index

### Comparison of parameters in Roussouly classification

More patients in NMC were Roussouly-type 1 compared to those in MC (*P* = 0.035). Compared to patients in MC, there were more patients in NMC matching ideal LA (*P* < 0.001). There were more patients who matched Roussouly-type in NMC compared with that in MC (*P* = 0.048). The Roussouly score in NMC was higher than that in MC (*P* = 0.032) (Table 3).

**Table 3:** Comparison of parameters in Roussouly classification between MC and NMC

Variables	MC (n = 41)	NMC (n = 39)	*P* value
Roussouly-type			0.082
1	4 (9.8%)	11 (28.2%)	0.035
2	5 (12.2%)	6 (15.4%)	0.679
3	23 (56.1%)	19 (48.7%)	0.509
4	9 (22.0%)	3 (7.7%)	0.074
Post-LA			0.262
L2	2 (4.9%)	0 (0%)	0.162
L2/3	3 (7.3%)	0 (0%)	0.085
L3	4 (9.8%)	6 (15.4%)	0.447
L3/4	3 (7.3%)	3 (7.7%)	0.949
L4	12 (29.3%)	16 (41.0%)	0.270
L4/5	11 (26.8%)	6 (15.4%)	0.211
L5	6 (14.6%)	8 (20.5%)	0.489
Ideal LA			0.082
L3/4	9 (30.0%)	3 (7.7%)	0.074
L4	23 (56.1%)	19 (48.7%)	0.509
L4/5	5 (12.2%)	6 (15.4%)	0.679
L5	4 (9.8%)	11 (28.2%)	0.035
Match ideal LA	9 (30.0%)	25 (64.1%)	< 0.001
Post-IP			0.033
T11	1 (2.4%)	0 (0%)	0.326
T12	8 (19.5%)	2 (5.1%)	0.053
L1	19 (46.3%)	15 (38.5%)	0.476
L1/2	0 (0%)	2 (5.1%)	0.142
L2	8 (19.5%)	18 (46.2%)	0.011
L3	5 (12.2%)	2 (5.1%)	0.264
Ideal IP			0.082
T12	9 (22.0%)	3 (7.7%)	0.074
L1	23 (56.1%)	19 (48.7%)	0.509
L2	5 (12.2%)	6 (15.4%)	0.679
L3	4 (9.8%)	11 (28.2%)	0.035
Match ideal Post-IP	15 (36.6%)	11 (28.2%)	0.424
Post-PI	53.1 ± 13.0	48.7 ± 8.9	0.082
Post-PT	25.0 ± 12.2	19.4 ± 5.3	0.009
Post-SS	27.7 ± 6.0	28.6 ± 8.3	0.577
Match Roussouly-type	3 (7.3%)	9 (23.1%)	0.048
Roussouly score	0.6 ± 0.6	0.9 ± 0.7	0.032

Post, postoperative; MC, mechanical complication group; NMC, no mechanical complication group; LA, lumbar apex; IP, inflexion point; PI, pelvic incidence; PT, pelvic tilt; SS, sacral slope

### Comparison of parameters in GAP score

The GAP score in MC was higher than that in NMC (*P* = 0.005). The postoperative (Post-) RPV score (P = 0.003) and Post-GT (*P* = 0.007) in MC were significantly higher than those in NMC. The Post-RPV (*P* = 0.019) and Post- RLL (*P* = 0.006) in MC were significantly lower than those in NMC. The number of patients with moderately disproportioned GAP score in NMC was more than that in MC (*P* = 0.010). There were more patients with severely disproportioned GAP score in MC compared with those in NMC (*P* = 0.003) (Table 4).

**Table 4:** Comparison of parameters in GAP score between MC and NMC

Post-variables	MC (n = 41)	NMC (n = 39)	*P* value
GAP score	8.8 ± 2.7	6.8 ± 3.4	0.005
Post-PI	53.1 ± 13.0	48.7 ± 8.9	0.082
Post-SS	27.7 ± 6.0	28.6 ± 8.3	0.577
Ideal SS	40.3 ± 7.7	37.7 ± 5.2	0.082
Post-RPV	− 12.6 ± 7.7	− 9.1 ± 5.1	0.019
Post-RPV score	2.0 ± 1.0	1.3 ± 1.1	0.003
Post-LL	32.3 ± 11.0	35.5 ± 10.2	0.177
Ideal LL	61.9 ± 8.1	59.2 ± 5.5	0.082
Post- RLL	− 29.7 ± 10.6	− 23.7 ± 8.0	0.006
Post-RLL score	2.5 ± 0.7	2.2 ± 0.9	0.054
Post-LDI	0.9 ± 0.3	0.8 ± 0.2	0.200
Post-LDI score	1.7 ± 1.5	1.3 ± 1.5	0.256
Post-GT	27.3 ± 13.5	20.6 ± 6.6	0.007
Post-Age	76.1 ± 2.3	76.9 ± 2.7	0.124
GAP score categories			0.012
Proportioned	1 (2.4%)	3 (7.7%)	0.281
Moderately disproportioned	7 (17.1%)	17 (43.6%)	0.010
Severely disproportioned	33 (80.5%)	19 (48.7%)	0.003

GAP score, global alignment and proportion score; Post, postoperative; MC, mechanical complication group; NMC, no mechanical complication group; SS, sacral slope; LL, lumbar lordosis; RPV, relative pelvic version; RLL, relative lumbar lordosis; LDI, lordosis distribution index; GT, global tilt

### Correlations between evaluation systems and mechanical complications

The results of logistic regression showed that: GAP score (P = 0.008) and GAP categories (*P* = 0.007) were positively correlated with mechanical complications; Roussouly score was negatively correlated with mechanical complications (*P* = 0.034); GAP score was positively correlated with PJK (*P* = 0.021); Roussouly score was negatively correlated with implant-related complications (*P* = 0.018); GAP categories were correlated with implant loosening (*P* = 0.023) (Table 5).

**Table 5:** Correlations between evaluation systems and mechanical complications

Characteristics	B value	SE	Wald value	*P* value	Exp (B) value	95% CI
Mechanical complications						
GAP score	1.602	0.079	7.103	0.008	1.233	(1.057, 1.439)
Contant	− 1.602	0.667	5.770	0.016	0.201	
GAP catergories	1.211	0.449	7.283	0.007	3.358	(1.393, 8.092)
Contant	− 1.910	0.779	6.017	0.014	0.148	
Roussouly score	− 0.721	0.341	4.481	0.034	0.486	(0.249, 0.948)
Contant	0.590	0.342	2.969	0.085	1.804	
Match Roussouly-type	− 0.668	0.355	3.536	0.060	0.513	(0.256, 1.029)
Contant	0.236	0.244	0.937	0.333	1.267	
PJK						
GAP score	0.656	0.283	5.362	0.021	1.927	(1.106, 3.357)
Contant	− 9.199	3.195	8.287	0.004	< 0.001	
Roussouly score	0.108	0.654	0.027	0.869	1.114	(0.309, 4.016)
Contant	− 2.792	0.696	16.071	< 0.001	0.061	
PJF						
GAP score	0.269	0.291	0.854	0.355	1.308	(0.740, 2.313)
Contant	− 6.081	2.981	4.161	0.041	0.002	
DJK or DJF						
GAP score	− 0.177	0.218	0.659	0.417	0.838	(0.547, 1.284)
Contant	− 2.431	1.504	2.614	0.106	0.088	
GAP catergories	− 1.349	1.003	1.808	0.179	0.259	(0.036, 1.854)
Contant	− 1.865	1.269	2.169	0.141	0.155	
Roussouly score	− 0.581	1.150	0.255	0.613	0.559	(0.059, 5.328)
Contant	− 3.301	0.923	12.787	< 0.001	0.037	
Implant-related complications						
GAP score	0.085	0.073	1.372	0.241	1.089	(0.944, 1.258)
Contant	− 0.979	0.627	2.443	0.118	0.376	
GAP catergories	0.573	0.416	1.897	0.168	1.774	(0.785, 4.010)
Contant	− 1.231	0.721	2.910	0.088	0.292	
Roussouly score	− 0.846	0.358	5.588	0.018	0.429	(0.213, 0.865)
Contant	0.301	0.337	0.801	0.371	1.352	
Match Roussouly-type	− 0.922	0.710	1.686	0.194	0.398	(0.099, 1.599)
Contant	-0.177	0.243	0.528	0.467	0.838	
Implant loosening						
GAP score	0.151	0.079	3.682	0.055	1.163	(0.997, 1.357)
Contant	− 1.730	0.694	6.219	0.013	0.177	
GAP catergories	1.127	0.495	5.180	0.023	3.087	(1.169, 8.147)
Contant	− 2.382	0.888	7.199	0.007	0.092	
Roussouly score	− 0.511	0.347	2.169	0.141	0.600	(0.304, 1.184)
Contant	− 0.144	0.333	0.187	0.665	0.866	
Match Roussouly-type	− 0.681	0.711	0.916	0.338	0.506	(0.126, 2.040)
Contant	− 0.418	0.248	2.841	0.092	0.659	
Implant breakage						
GAP score	− 0.260	0.165	2.496	0.114	0.771	(0.558, 1.065)
Contant	− 1.219	1.037	1.380	0.240	0.296	
GAP catergories	− 1.425	0.748	3.628	0.057	0.241	(0.056, 1.042)
Contant	− 1.031	0.961	1.150	0.284	0.357	

SE, standard error; CI, confidence interval; GAP score, global alignment and proportion score; PJK, proximal junctional kyphosis; PJF, proximal junctional failure; DJK, distal junctional kyphosis; DJF, distal junctional failure

### ROC of evaluation systems in predicting mechanical complications

**Fig. 1:**
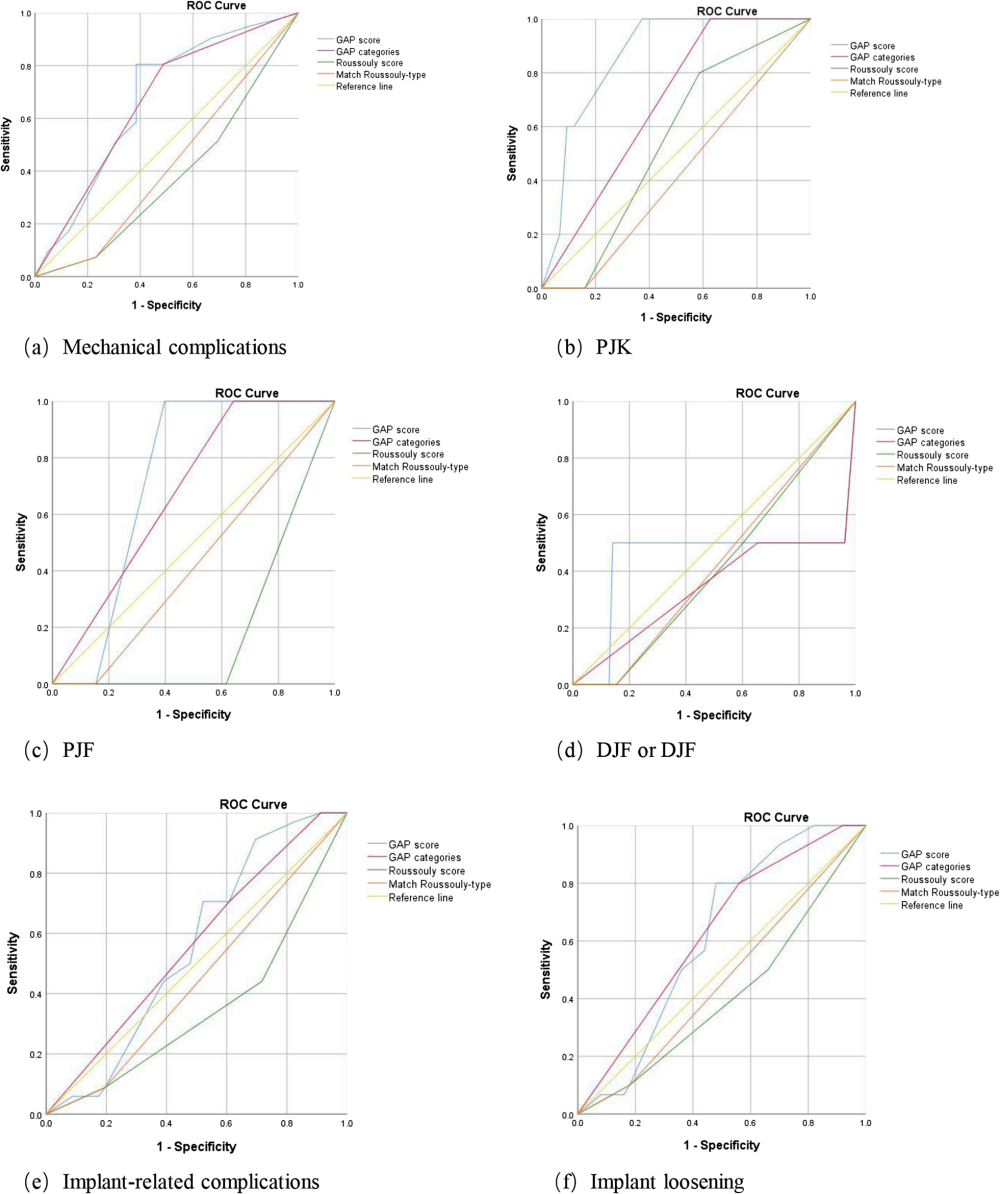
ROC curve of evaluation systems in predicting **a** Mechanical complications, **b** PJK, **c** PJF, **d** DJK or DJF, **e** Implant-related complications and **f** Implant loosening

Results of ROC showed that GAP score was more effective in predicting mechanical complications than the Roussouly classification (Fig. 1). GAP score (Cut-off value = 10) was more effective in predicting PJK (AUC = 0.863) and PJF (AUC = 0.724) than Roussouly score. GAP categories (AUC = 0.561, Cut-off value = Severely disproportioned) was more effective than GAP score (AUC = 0.555, Cut-off value = 5) in predicting implant-related complications (Table 6).

**Table 6:** Results of ROC analyzing evaluation systems in predicting mechanical complications

Characteristics	AUC	Cut-off value	Sensitivity	1-Specificity	Youden index
Mechanical complications					
GAP score	0.669	8	0.805	0.385	0.420
GAP catergories	0.660	Moderately disproportioned	0.976	0.923	0.053
PJK					
GAP score	0.863	10	1.000	0.373	0.627
GAP catergories	0.687	Severely disproportioned	1.000	0.627	0.373
Roussouly score	0.543	1	0.800	0.587	0.213
PJF					
GAP score	0.724	10	1.000	0.397	0.603
GAP catergories	0.679	Severely disproportioned	1.000	0.641	0.359
DJK or DJF					
GAP score	0.442	11	0.500	0.141	0.359
Implant-related complications					
GAP score	0.555	5	0.912	0.696	0.216
GAP catergories	0.561	Severely disproportioned	0.706	0.609	0.097
Implant loosening					
GAP score	0.615	8	0.800	0.480	0.320
GAP catergories	0.628	Severely disproportioned	0.800	0.560	0.24
Implant breakage					
GAP score	0.217	8	1.000	0.947	0.053

ROC, receiver operator characteristic curve; AUC, area under the curve; GAP score, global alignment and proportion score; PJK, proximal junctional kyphosis; PJF, proximal junctional failure; DJK, distal junctional kyphosis; DJF, distal junctional failure

## Discussion

The most common mechanical complication in this study was screw loosening. This was due to a decrease in bone density in older patients which made these patients more sensitive to postoperative sagittal imbalance [6]. Lumbar degeneration and thoracolumbar coronal deformity could modify lumbar lordosis, which could consequently influence SS [8]. Therefore, SS becomes an inadequate parameter to classify sagittal types in pathologic patients. In addition, the Roussouly classification relies on PI which is considered not to vary with age, pathology, or compensation [15]. However, Roussouly classification is based on the classification of normal spine; most of the studies related to the compensatory mechanism of spinal degeneration were cross-sectional studies [16–18]. In this study, more cases without mechanical complications were Roussouly-type 1 compared to those with mechanical complications. This was because Roussouly-type 1 is a combination of long kyphosis and short lordosis at the lower arc of the spine. Inflexion point, which represents the region with the highest junctional stress concentration, has already been fixed in the central structure of the long-segment internal fixation system [19]. Our study showed: there were more patients who matched Roussouly-type in the no mechanical complication group compared with that in mechanical complication groups; compared to cases with mechanical complications, there were more patients without mechanical complications matching ideal LA. These results suggested that the difference in Roussouly type matching between the two groups was mainly due to the ideal LA matching, but not the ideal IP matching. Changing the original IP of the spine can easily lead to overcorrection of spinal deformities, thus increasing the stress on the internal fixation system and then the risk of mechanical complications. Therefore, it appeared to be more important to adjust LA of ADS patients during surgery. The current study showed: there was no significant correlation between Roussouly-type matching and mechanical complications; the ROC analysis implied that Roussouly-type matching could not accurately predict the risk of mechanical complications. Roussouly-type only morphologically described the sagittal characteristics of ADS patients, which lacked three-dimensional analysis and quantitative indicators of the spinal deformity in ADS patients.

In the current study, GAP score was better than Roussouly classification in predicting mechanical complications, PJK and PJF. However, the prediction accuracy of GAP score for implant breakage and DJK or DJF was low. This was because implant breakage is closely related to the material properties (elastic modulus and Poisson’s ratio) of the internal fixation system itself, the living habits of patients, and the overall structure (shape features and spatial structures) of the internal fixation [7]. The occurrence of DJK is affected by many factors, such as the distal fixation method, the severity of ADS, and the levels of internal fixation; these factors are not fully reflected in the GAP score, so the accuracy of prediction is also low [18].

There are some limitations in this study. Firstly, because older patients are more sensitive to spinal sagittal imbalance, the patients included in this study were older than those in previous studies, which could introduce a selection bias. Secondly, this study only analyzed the parameters involved in Roussouly classification and GAP score, while did not assess the conditions of paraspinal muscles and lower limb compensations. This prevented the results of this study from explaining all the causes of postoperative mechanical complications.

## Conclusion

Both Roussouly classification and GAP score used PI-based sagittal parameters to quantify the shape and alignment of the sagittal plane. Roussouly classification could only be a rough estimate of optimal spinopelvic alignment which includes the restoration of ideal LL, ideal pelvic version, and ideal lordosis distribution. Quantitative parameters in GAP score made it more effective in predicting mechanical complications than Roussouly classification. In the prediction of mechanical complications, GAP score was more effective in predicting PJK and PJF. Therefore, GAP score should play an important role in preoperative evaluation.

## Supplementary Information

**Additional file 1. Supplementary file 1**. The definitions of radiographic parameters.

**Additional file 2. Supplementary file 2**. Ideal values of different parameters in Roussouly classification.

**Additional file 3. Supplementary file 3**. Cut-off values of the GAP score.

## Abbreviations

ADS: Adult degenerative scoliosis
AUC: Area under the curve
CA: Coronal Cobb angle
CI: Confidence interval
DJF: Distal junctional failure
DJK: Distal junctional kyphosis
GAP: Global Alignment and Proportion
GT: Global tilt
IP: Inflexion point
JOA: Japanese Orthopaedic Association
LA: Lumbar sagittal apex
LDI: Lordosis distribution index
LIV: Lower instrumented vertebra
LL: Lumbar lordosis
NVL: The number of vertebrae included in the lordosis
ODI: Oswestry Disability Index
OR: Odds ratio
PI: Pelvic incidence
PJK: Proximal junctional kyphosis
PJF: Proximal junctional failure
PLL: Relative lumbar lordosis
PT: Pelvic tilt
ROC: Receiver operating characteristic
RPV: Relative pelvic version
RSA: Relative spinopelvic alignment
SRS: Scoliosis Research Society
SS: Sacral slope
SVA: Sagittal vertical axis
TK: Thoracic kyphosis
TLK: Thoracolumbar kyphosis
UIV: Upper instrumented vertebra
VAS: Visual analogue scale
