# Supplementary material for: Which sagittal evaluation system can effectively predict mechanical complications in the treatment of elderly patients with adult degenerative scoliosis? Roussouly classification or Global Alignment and Proportion (GAP) Score
Source: J Orthop Surg Res. 2021 Oct 26;16:641. doi: 10.1186/s13018-021-02786-8 (PMC8549320; doi:10.1186/s13018-021-02786-8)
Supplement: Supplementary file 1 — Additional file 1. Supplementary file 1. The definitions of radiographic parameters. [file 13018_2021_2786_MOESM1_ESM.docx]

| **Supplementary file 1** The definitions of radiographic parameters | |
| --- | --- |
| Radiographic parameters | Definitions |
| Pelvic incidence, PI | The angle between the line, which connects the midpoint of the superior endplate of the sacrum and the midpoint of the femoral head, and the vertical line of the superior endplate of the sacrum. |
| Pelvic tilt, PT | The angle between the line, which connects the midpoint of the superior endplate of the sacrum and the midpoint of the femoral head, and the plumb line of the midpoint of the superior endplate of the sacrum. |
| Sacral slope, SS | The Cobb angle between the upper endplate of the sacrum and the horizontal line. |
| Thoracolumbar kyphosis, TLK | The Cobb angle between the upper endplate of T4 and the lower end plate of T12. |
| Thoracic kyphosis, TK | The Cobb angle between the upper endplate of T4 and the lower end plate of T12 |
| Lumbar lordosis, LL | The Cobb angle between the upper endplate of L1 and the upper end plate of S1. |
| L4-S1 lordosis | The Cobb angle between the upper endplate of L4 and the upper end plate of S1. |
| Global tilt, GT | The Cobb angle between the line，which connects the geometric center of C7 and the center of the upper endplate of the sacrum, and the line, which is between the upper endplate of the sacrum and the midpoint of the femoral head. |
| Sagittal vertical axis, SVA | The distance from the C7 plumb line to the posterior upper corner of the sacrum. |
| NVL | The number of vertebrae included in the lordosis. |
| Lumbar sagittal apex, LA | The apex divides upper and lower lumbar arches. |
| Inflexion point, IP | The cranial vertebra in the transition between lordosis and kyphosis. |
