# Supplementary material for: Which sagittal evaluation system can effectively predict mechanical complications in the treatment of elderly patients with adult degenerative scoliosis? Roussouly classification or Global Alignment and Proportion (GAP) Score
Source: J Orthop Surg Res. 2021 Oct 26;16:641. doi: 10.1186/s13018-021-02786-8 (PMC8549320; doi:10.1186/s13018-021-02786-8)
Supplement: Supplementary file 2 — Additional file 2. Supplementary file 2. Ideal values of different parameters in Roussouly classification. [file 13018_2021_2786_MOESM2_ESM.docx]

| **Supplementary file 2** Ideal values of different parameters in Roussouly classification | | | | | | | |
| --- | --- | --- | --- | --- | --- | --- | --- |
| Roussouly-type | PI | SS | LL | NVL | LDI (%) | Lumbar Apex | Inflexion point |
| 1 | < 45° | < 35° | 45° | 3 | 90 | L5 | L3 |
| 2 | < 45° | < 35° | 50° | 4 | 80 | L4/5 disk | L2 |
| 3 | 45°–60° | 35–45° | 55° | 5 | 70 | L4 | L1 |
| 4 | > 60° | > 45° | 60° | 6 | 60 | L3/4 disk | T12 |
| Notice: PI, pelvic incidence; SS, sacral slope; LL, lumbar lordosis; NVL, number of vertebrae in the lordosis; LDI, lordosis distribution index. | | | | | | | |
