# Supplementary material for: Which sagittal evaluation system can effectively predict mechanical complications in the treatment of elderly patients with adult degenerative scoliosis? Roussouly classification or Global Alignment and Proportion (GAP) Score
Source: J Orthop Surg Res. 2021 Oct 26;16:641. doi: 10.1186/s13018-021-02786-8 (PMC8549320; doi:10.1186/s13018-021-02786-8)
Supplement: Supplementary file 3 — Additional file 3. Supplementary file 3. Cut-off values of the GAP score. [file 13018_2021_2786_MOESM3_ESM.docx]

| **Supplementary file 3** Cut-off values of the GAP score | | | | | |
| --- | --- | --- | --- | --- | --- |
| Score | RPV | RLL | LDI | RSA | Age |
| 3 | Severe retroversion,  ＜-15° | Severe hypolordosis,  ＜-25°;  Hyperlordosis, ＞11° | Hyperlordotic maldistribution,  ＞80% | Severe positive malalignment,  ＞18° | - |
| 2 | Moderate retroversion,  -15°to-7.1° | Moderate hypolordosis, -25° to -14.1° | Severe hypolordotic maldistribution, ＜40% | - | - |
| 1 | Anteversion,  ＞5° | - | Moderate hypolordotic maldistribution,  40% to 49% | Moderate positive malalignment,  10.1° to 18°;  Negative malalignment,  ＜-7° | ≥60years |
| 0 | Aligned, -7° to 5° | Aligned, -14° to 11° | Aligned, 50% to 80% | Aligned, 10° to -7° | ＜60years |
|  |  | GAP Score Categories | |  |  |
|  | Proportioned | Moderately disproportioned | | Severely disproportioned | |
| Total score: | 0-2 | 3-6 | | ≥7 | |
| Notice: GAP, global alignment and proportion; RPV, relative pelvic version; RLL relative lumbar lordosis; LDI lordosis distribution index; RSA, relative spinopelvic alignment. | | | | | |
